# Two Hematological Markers Predicting the Efficacy and Prognosis of Neoadjuvant Chemotherapy Using Lobaplatin Against Triple-Negative Breast Cancer

**DOI:** 10.1093/oncolo/oyae025

**Published:** 2024-03-02

**Authors:** Cheng Wang, Qiyun Shi, Guozhi Zhang, Xiujuan Wu, Wenting Yan, Andi Wan, Siyi Xiong, Long Yuan, Hao Tian, Dandan Ma, Jun Jiang, Xiaowei Qi, Yi Zhang

**Affiliations:** Department of Breast and Thyroid Surgery, Southwest Hospital, Army Medical University, Chongqing, People’s Republic of China; Department of Breast and Thyroid Surgery, Southwest Hospital, Army Medical University, Chongqing, People’s Republic of China; Department of Breast and Thyroid Surgery, Southwest Hospital, Army Medical University, Chongqing, People’s Republic of China; Department of Breast and Thyroid Surgery, Southwest Hospital, Army Medical University, Chongqing, People’s Republic of China; Department of Breast and Thyroid Surgery, Southwest Hospital, Army Medical University, Chongqing, People’s Republic of China; Department of Breast and Thyroid Surgery, Southwest Hospital, Army Medical University, Chongqing, People’s Republic of China; Department of Breast and Thyroid Surgery, Southwest Hospital, Army Medical University, Chongqing, People’s Republic of China; Department of Breast and Thyroid Surgery, Southwest Hospital, Army Medical University, Chongqing, People’s Republic of China; Department of Breast and Thyroid Surgery, Southwest Hospital, Army Medical University, Chongqing, People’s Republic of China; Department of Breast and Thyroid Surgery, Southwest Hospital, Army Medical University, Chongqing, People’s Republic of China; Department of Breast and Thyroid Surgery, Southwest Hospital, Army Medical University, Chongqing, People’s Republic of China; Department of Breast and Thyroid Surgery, Southwest Hospital, Army Medical University, Chongqing, People’s Republic of China; Department of Breast and Thyroid Surgery, Southwest Hospital, Army Medical University, Chongqing, People’s Republic of China

**Keywords:** PLR, NLR, TNBC, lobaplatin, tpCR, survival

## Abstract

**Background:**

Our previous work indicated that the addition of lobaplatin to combined therapy with taxane and anthracycline can improve the pathological complete response rate of neoadjuvant therapy for triple-negative breast cancer (TNBC) and lengthen long-term survival significantly, but the therapeutic markers of this regimen are unclear.

**Methods:**

Eighty-three patients who met the inclusion criteria were included in this post hoc analysis. We analyzed the association between platelet-to-lymphocyte ratio (PLR) and neutrophil-to-lymphocyte ratio (NLR) before neoadjuvant chemotherapy with the efficacy and prognosis after treatment with docetaxel, epirubicin, and lobaplatin neoadjuvant chemotherapy regimen. χ^2^ test and Cox regression were used to analyze the association between PLR and NLR with total pathologic complete response (tpCR), as well as the association between PLR and NLR with event-free survival (EFS) and overall survival (OS), respectively.

**Results:**

The tpCR rate in the PLR− group was 49.0% (25/51), which was significantly higher than that in the PLR+ group (25.0% [8/32], *P* = .032). The tpCR rate in the NLR− group was 49.1% (26/53), which was significantly higher than that in the NLR+ group (23.3% [7/30], *P* = .024). The tpCR rate of the PLR−NLR− (PLR− and NLR−) group was 53.7% (22/41), which was significantly higher than that of the PLR+/NLR+ (PLR+ or/and NLR+) group (26.1% [11/42]; *P* = .012). EFS and OS in the NLR+ group were significantly shorter than those in the NLR− group (*P* = .028 for EFS; *P* = .047 for OS). Patients in the PLR−NLR− group had a longer EFS than those in the PLR+/NLR+ group (*P* = .002).

**Conclusion:**

PLR and NLR could be used to predict the efficacy of neoadjuvant therapy with the taxane, anthracycline, and lobaplatin regimen for patients with TNBC, as patients who had lower PLR and NLR values had a higher tpCR rate and a better long-term prognosis.

## Teaser

To clarify the predictive effect on the efficacy of therapy, this study analyzed the relationship between platelet-to-lymphocyte ratio and neutrophil-to-lymphocyte ratio before neoadjuvant chemotherapy and the effect of neoadjuvant chemotherapy with lobaplatin for triple-negative breast cancer.

**Implications for Practice**

The addition of lobaplatin to combined therapy with taxane and anthracycline can improve the pathological complete response rate of neoadjuvant therapy for triple-negative breast cancer (TNBC) and lengthen long-term survival significantly, but the therapeutic markers of this regimen are unclear. This study reports for the first time the independent predictive value of platelet/lymphocyte ratio and neutrophil/lymphocyte ratio for early TNBC treated with a neoadjuvant regimen comprising taxane and anthracycline combined with lobaplatin and provides further reference for the selection of clinical chemotherapy regimens, avoiding the side effect of chemotherapy, and preventing the occurrence of disease progression.

## Introduction

Triple-negative breast cancer (TNBC) accounts for 10%-20% of all breast cancers.^1^ It has high recurrence and metastasis rates and has the worst prognosis among the known breast cancer subtypes.^2^ There are no clear treatment targets for this cancer, and the treatment options are limited. Currently, neoadjuvant therapy is the standard treatment for patients with early-stage TNBC who have lymph node-positive tumors or tumors that are >2 cm in size. Pathologic complete response (pCR) is an important indicator of prognosis, as the long-term survival of patients with TNBC who achieve pCR after neoadjuvant therapy is better than that of patients with invasive tumor residue.^3^ Therefore, strategies for the optimization of therapeutic regimens to improve the prognosis of TNBC should focus on improving the rate of pCR.

For a long time, combined or sequential treatment with anthracycline and taxane has been the standard regimen of neoadjuvant chemotherapy for TNBC. In recent years, a number of studies have added platinum to this standard regimen, and this has significantly increased the pCR rate and further improved the survival of patients. However, the addition of platinum also resulted in more treatment-related adverse events.^4^ In previously published studies by our group, the addition of lobaplatin, a water-soluble platinum compound, to the standard regimen was found to significantly improve the curative effect and prognosis of TNBC. However, some patients still do not show significant benefits and exhibit a greater number of treatment-related side effects with the addition of lobaplatin.^5,6^ Therefore, identifying biomarkers that can be used to predict the effect and prognosis of neoadjuvant chemotherapy with lobaplatin, in order to improve treatment accuracy for TNBC, is an important topic of focus.

The role of blood cells in cancer pathogenesis and progression is well known. For example, mechanistic studies have shown that platelets may promote tumor development by expressing factors, such as GRTIL, TACI, and ADAM17,^7-9^ and helping to create a microenvironment that is conducive to the development of primary and metastatic tumors.^10^ Accordingly, it has also been demonstrated that metastasis of breast cancer to the bone and lung can be prevented by blocking platelets.^11^ Moreover, neutrophils may promote tumor metastasis and development through neutrophil extracellular traps.^12^ The predictive effect of specific blood cell types on the short-term efficacy and long-term prognosis of neoadjuvant chemotherapy for patients with cancer is well known, mainly because of their close relationship with the inflammatory/immune environment. In patients with malignant tumors, such as liver cancer, endometrial carcinoma, renal cell carcinoma, and breast cancer, platelet-to-lymphocyte ratio (PLR) and neutrophil-to-lymphocyte ratio (NLR) are known as good predictors of prognosis, and high PLR and NLR values are often significantly correlated with poor prognosis.^13-16^ With regard to prognosis prediction for chemotherapy regimens, combination treatment with 5-fluorouracil, epirubicin, and cyclophosphamide was found to be associated with a significantly better pCR rate, disease-free survival (DFS), and overall survival (OS) in patients with TNBC with low NLR than in those with high NLR.^17^ Further, in the case of the doxorubicin and cyclophosphamide regimen with sequential docetaxel, NLR was able to predict the pCR rate, as the pCR rate and 5-year recurrence-free survival (RFS) were significantly better in patients with TNBC with low NLR than in those with high NLR.^18^ In the case of TNBC, taxane and anthracycline chemotherapy resulted in a significantly higher pCR rate in patients with low PLR than in patients with high PLR.^19^ Further, a recent meta-analysis showed that high PLR was closely associated with poor prognosis, lymph node metastasis, and distant metastasis in breast cancer.^20^ These findings indicate that PLR and NLR are closely related to the prognosis of TNBC and can predict the efficacy of neoadjuvant chemotherapy with taxane, anthracycline, fluorouracil, and other drugs. However, to the best of our knowledge, no study so far has investigated whether NLR and PLR can be used to predict the efficacy and prognosis of neoadjuvant chemotherapy with lobaplatin in patients with TNBC. Therefore, based on the findings of previous clinical studies on other cancers, we analyzed the relationship between PLR and NLR before neoadjuvant chemotherapy and the effect and prognosis of neoadjuvant chemotherapy with lobaplatin for TNBC, in order to clarify their predictive effect on the efficacy of therapy and provide a reference for the precise application of the platinum neoadjuvant regimen in patients with TNBC.

## Methods

### Study Design and Participants

We performed a post hoc analysis of a randomized controlled clinical study about docetaxel combined with epirubicin and lobaplatin as a neoadjuvant chemotherapy regimen for TNBC. Relevant research results have been reported previously.^5,6^ From January 2014 to August 2019, 83 patients were included in this study. All patients were enrolled based on the following inclusion criteria: (1) age = 18-70 years, (2) Eastern Cooperative Oncology Group (ECOG) score = 0 or 1, (3) pathologically confirmed stages I-III TNBC, (4) no treatment before neoadjuvant therapy, (5) no history of other cancers, bilateral breast cancer, or inflammatory breast cancer, and (6) no systemic diseases, acute and chronic inflammatory diseases, or autoimmune diseases. In all the included cases, the triple-negative status was histologically confirmed based on <1% ER and PR expression, determined by local immunohistochemical (IHC) analysis, and absence of HER2 (IHC score 0 to 1+, or IHC score 2+ with no amplification according to the results of in situ hybridization).

### Post Hoc Analysis

The primary objective of the current study was to estimate the association between PLR and NLR with total pathologic complete response (tpCR) and event-free survival (EFS). TpCR is defined as the pathologic complete response of the breast and axilla at the same time. EFS was defined as the time from enrollment to the date of disease progression that precluded definitive surgery, local or distant recurrence, occurrence of a second primary cancer, or death from any cause, whichever occurred first. The secondary objective was to estimate the association between PLR and NLR with OS. OS was defined as the time from enrollment to death from any cause.

### Data Collection

We recorded each patient’s baseline characteristics based on breast ultrasound, tumor biopsy, and laboratory tests. Blood test results before the first cycle of neoadjuvant chemotherapy were collected to calculate PLR and NLR. We divided patients into the low PLR group (PLR−), high PLR group (PLR+), low NLR group (NLR−), and high NLR group (NLR+) by the mean values of PLR and NLR. Before surgery, all the patients received 4 cycles of a chemotherapy regimen that comprised 75 mg/m^2^ docetaxel (T), 80 mg/m^2^ epirubicin (E), and 30 mg/m^2^ of lobaplatin (L), and also received 2 cycles of treatment with the same regimen after surgery. TpCR status was recorded based on the post-operative pathology report. Endpoint events were recorded based on follow-up results.

### Statistical Analysis

SPSS 26.0 (IBM Corp., Armonk, NY, USA) was used to analyze the data. The χ^2^ test or Fisher’s exact test was used to evaluate baseline differences between PLR− group with PLR+ group, as well as between NLR+ group with NLR− group, and the χ^2^ test was used to analyze the association between the tpCR rate with PLR and NLR. Univariate and multivariate Cox proportional risk regression models were used to explore the associated factors of EFS and OS. The Kaplan-Meier curve drawn by R 4.2.2 and the log-rank test were used to perform survival analyses and to evaluate the association between PLR and NLR with EFS and OS. Bilateral *P* values <.05 were considered statistically significant.

## Results

### Screening of Patients

**Figure 1.**
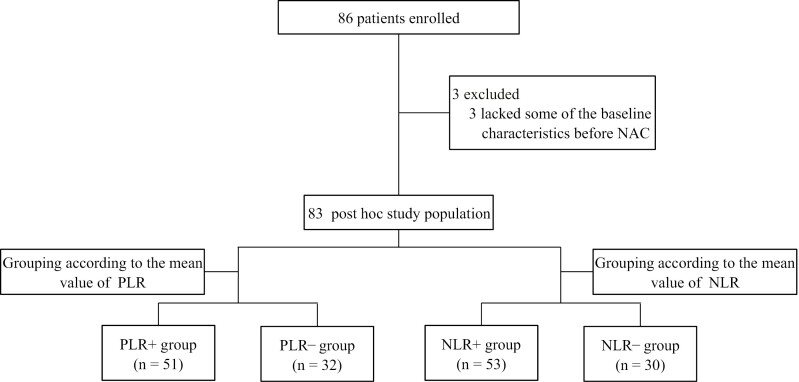
Trial profile and selection process of the post hoc study population. Abbreviations: PLR−, low PLR; PLR+, high PLR; NLR−, low NLR; NLR+, high NLR group.

Eighty-six patients with TNBC were included in the screening. Three patients were excluded because they lacked some of the baseline characteristics before NAC, ultimately, 83 patients were included in this trial. The mean values of PLR and NLR were 145.71 and 2.74, respectively. Based on the mean values of PLR and NLR, we divided patients into the PLR− group (*n* = 51), PLR+ group (*n* = 32), NLR− group (*n* = 53), and NLR+ group (*n* = 30; Figure 1).

### Clinicopathological Characteristics of the Patients

Between February 2014 and August 2019, 83 eligible female patients participated in the study. The average age of the patients was 46.5 years. The relatively young age of the selected patients was attributable to the inclusion criteria, which limited the cohort to patients who were <70 years old, as well as the early-onset age of breast cancer in Chinese women (with the peak being at approximately 50 years). There was no statistically significant difference between the groups in terms of age, menopausal status, T stage, N stage, clinical stage, Ki67 index, Her2 expression, and other clinicopathological characteristics. The clinicopathological characteristics of all the patients are summarized in Table 1.

**Table 1.** Summary of the clinicopathological characteristics of the cohort (*N* = 83).

Characteristics	PLR		*P* value	NLR		*P* value
	PLR−	PLR+		NLR−	NLR+	
Age						
<45	22	14	.997	23	13	.679
45-55	24	15		26	13	
>55	5	3		4	4	
Menopausal status						
Premenopausal	37	22	.700	41	18	.253
Perimenopausal	3	1		2	2	
Postmenopausal	11	9		10	10	
T stage						
T1-T2	47	26	.173	49	24	.186
T3-T4	4	6		4	6	
N stage						
Negative	22	17	.375	28	11	.156
Positive	29	15		25	19	
Clinical stage						
I	8	5	.994	10	3	.451
II-III	43	27		43	27	
Ki67						
<15%	8	4	.627	6	6	.366
15%-30%	14	12		19	7	
>30%	29	16		28	17	
HER2						
0	44	22	.054	43	23	.628
1+/2+	7	10		10	7	
ALL	51	32		53	30	

### Treatment Effect

**Figure 2.**
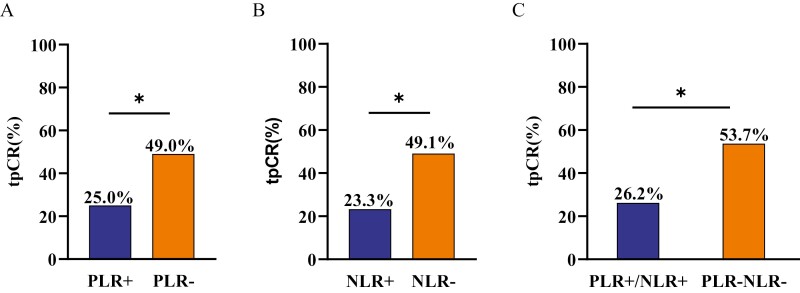
Comparison of the complete pathological response rate between groups. (**A**) PLR− versus PLR+, (**B**) NLR− versus NLR+, (**C**) PLR−/NLR− versus PLR+/NLR+.

The tpCR rate of the PLR− group was 49.0% (25/51), which was significantly higher than that of PLR+ group (25.0% [8/32]; OR = 2.885, 95% CI, 1.093-7.612, *P* = .032; Fig. 2A), and the tpCR rate of the NLR− group was 49.1% (26/53), which was also significantly higher than that of NLR+ group (23.3% [7/30], OR = 3.164, 95% CI, 1.161-8.626, *P* = .024; Fig. 2B). Further, the tpCR rate of the PLR−NLR− (PLR− and NLR−) group was 53.7% (22/41), which was significantly higher than that of the PLR+/NLR+ (PLR+ or/and NLR+) group (26.1% [11/42]; OR = 3.263, 95% CI, 1.298-8.204, *P* = .012; Fig. 2C). The tpCR rate of PLR−NLR− group, PLR−NLR+ group, PLR+NLR− group, and PLR+NLR+ group was 53.7% (22/41), 30.0% (3/10), 33.3% (4/12), and 20% (4/20), respectively (Supplementary Fig. S1 depicts the tpCR rate of the subgroups.).

### Survival Analysis

**Figure 3.**
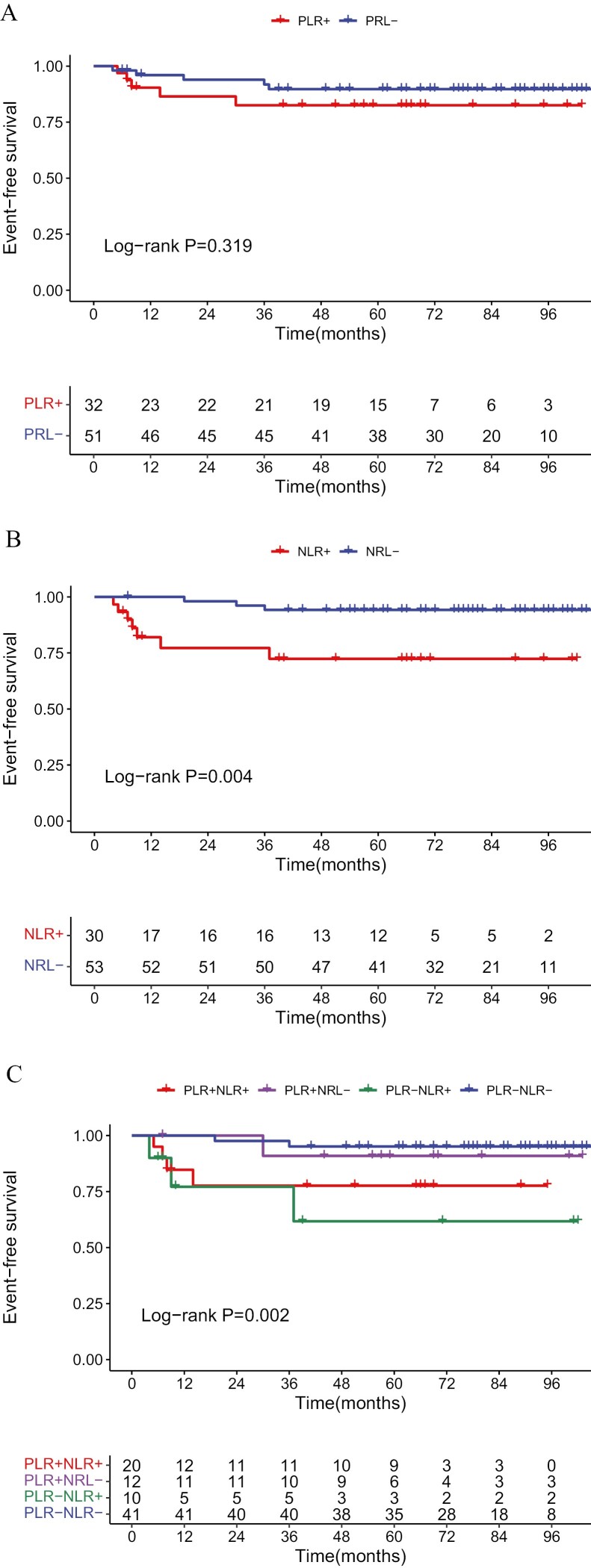
Comparison of EFS rate between groups. (**A**) PLR− versus PLR+; (**B**) NLR− versus NLR+; (**C**) PLR−NLR− versus PLR−NLR+ versus PLR+NLR− versus PLR+NLR+.

The median follow-up time was 69 months (4-106 months). The primary endpoint, ie, EFS, of the PLR− group was better than that of the PLR+ group, but the difference was not statistically significant (*P* = .319; Fig. 3A). In contrast, the EFS of the NLR− group was significantly better than that of NLR+ group (*P* = .004; Fig. 3B). The 5-year EFS rates of the PLR−NLR−, PLR+NLR−, PLR+NLR+, and PLR−NLR+ groups were 95.1%, 91.0%, 76.0%, and 59.1%, respectively. Compared with the PLR+/NLR+ group, the PLR−NLR− group had significantly longer EFS (*P* = .002; Fig. 3C).

**Figure 4.**
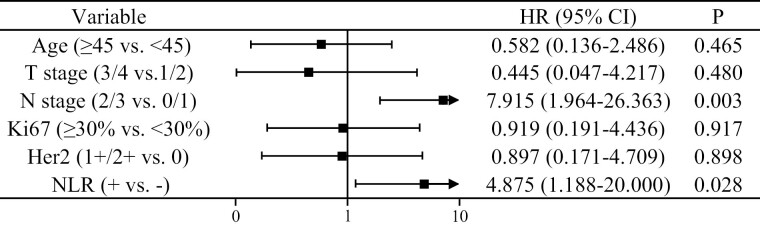
Multivariate analysis of factors independently associated with EFS. NLR and N stage were identified as significant predictors of EFS.

At first, we performed univariate and multivariate analyses of EFS. The results of the univariate analysis showed that there was no significant difference between the PLR+ group with the PLR− group (HR = 1.861, 95% CI, 0.537-6.443, *P* = .327), and the EFS of NLR− group was significantly longer than the NLR+ group (HR = 5.946, 95% CI, 1.526-23.169, *P* = .010; Supplementary Table S1). Next, we analyzed the correlation between PLR and NLR. The result of the analysis showed that the Pearson correlation coefficient between PLR and NLR was 0.618, *P* < .001. To avoid collinearity between PLR with NLR, only NLR was included in the multivariate analysis. As shown in Fig. 4, multivariate analysis showed that both NLR and N stage played a significant role in predicting prognosis. Specifically, the EFS of the N2/3 stage was worse than the N0/1 stage (HR = 7.915, 95% CI, 1.964-26.363, *P* = .003), and the EFS of the NLR+ group was worse than the NLR− group (HR = 4.875, 95% CI, 1.188-20.000, *P* = .028).

**Figure 5.**
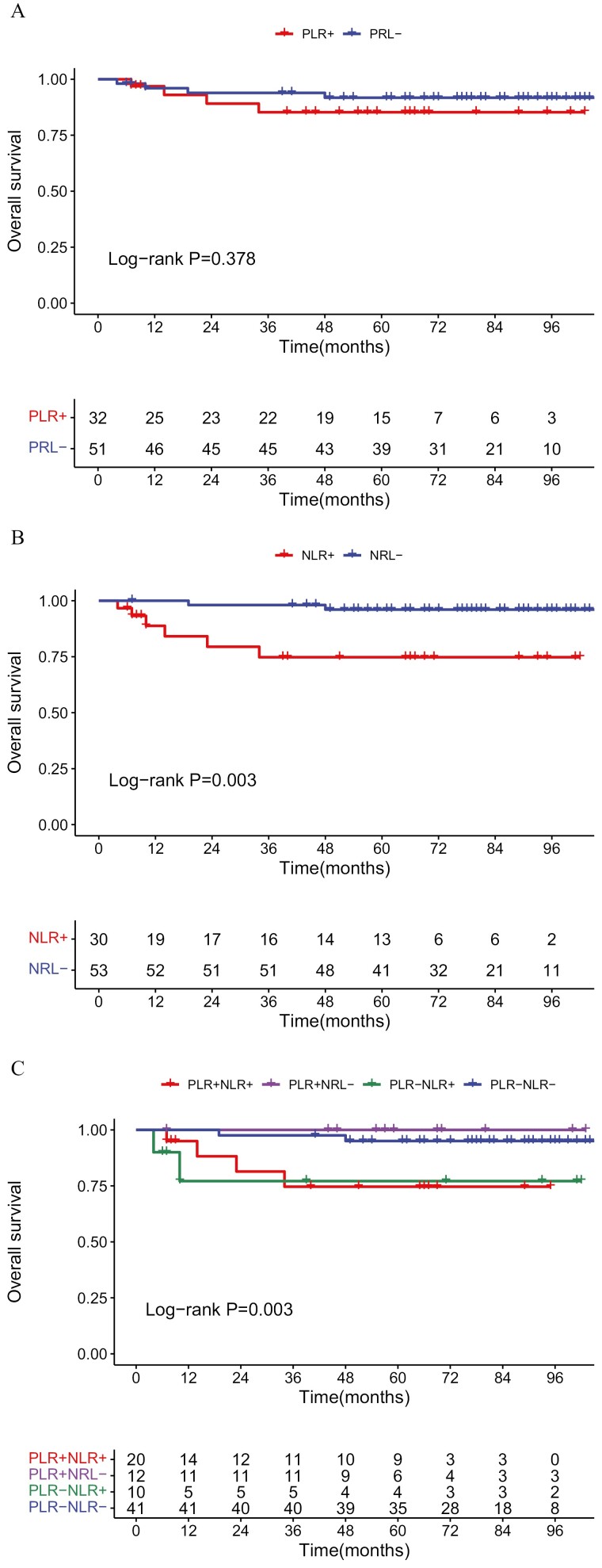
Comparison of OS between groups. (**A**) PLR− versus PLR+; (**B**) NLR− versus NLR+; (**C**) PLR−NLR− versus PLR−NLR+ versus PLR+NLR− versus PLR+NLR+.

Next, we analyzed differences in OS between subgroups. The OS of the PLR− group was better than that of PLR+ group, but the difference was not statistically significant (*P* = .378; Fig. 5A). However, the EFS of the NLR− group was significantly better than that of the NLR+ group (*P* = .003; Fig. 5B). When the 2 indicators were combined, the PLR−NLR− group was found to have a good OS (*P* = .003; Fig. 5C).

**Figure 6.**
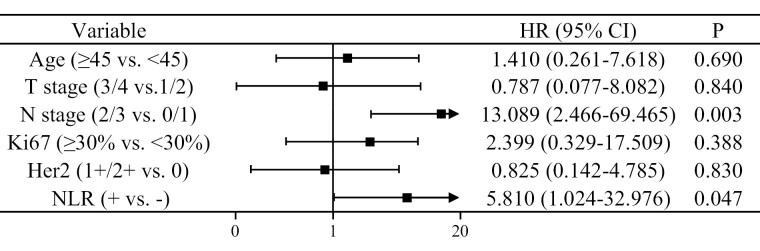
Multivariate analysis of factors independently associated with OS. N stage and NLR were identified as significant independent predictors of OS.

We also performed univariate and multivariate analyses of OS. The results of the univariate analysis showed that there was no significant difference in OS between the PLR+ group with the PLR− group (HR = 1.850, 95% CI, 0.462-7.414, *P* = .385), and the OS of NLR− group was significantly longer than the NLR+ group (HR = 7.803, 95% CI, 1.565-38.907, *P* = .012; Supplementary Table S2). To avoid collinearity between PLR with NLR, only NLR was included in the multivariate analysis. Multivariate analysis showed that NLR and N stage still played an important role in predicting OS, and the N2/3 stage was associated with a worse OS than the N0/1 stage (HR = 13.089, 95% CI, 2.466-69.465, *P* = .003). Further, the OS of the NLR+ group was worse than the NLR− group (HR = 5.810, 95% CI, 1.024-32.976, *P* = .047; Fig. 6).

## Discussion

In our previous series of studies, it was confirmed that the addition of lobaplatin can effectively improve the treatment effect of chemotherapy for TNBC and significantly improve the prognosis.^5,6^ The present study aims to examine NLR and PLR as biomarkers of the docetaxel, epirubicin, and lobaplatin (TEL) chemotherapy scheme for TNBC. The findings demonstrate, for the first time, that PLR and NLR, 2 hematological indicators that are easy to assess, can be used to predict the efficacy and prognosis of the TEL regimen for neoadjuvant treatment of TNBC.

The present cohort was divided into various subgroups based on the mean PLR and NLR values, and the findings indicated that the tpCR rate of patients with TNBC who underwent the TEL neoadjuvant chemotherapy regimen was significantly higher in the PLR− group, NLR− group, and PLR−NLR− group. In accordance with these results, another study on 120 cases of TNBC found that patients with PLR values lower than 133.25 had a significantly higher chance of achieving pCR after anthracycline/taxane-based neoadjuvant treatment.^21^ The present findings are also in agreement with previous studies on other cancer types. For example, in a cohort of 60 patients with esophageal cancer who received neoadjuvant treatment, high PLR and high NLR were predictive of poor tpCR rate.^22^ Further, a study of 86 cases of rectal cancer reported that NLR and PLR can be used as predictors of the pCR rate, with the pCR rate of groups with low NLR and PLR values being significantly higher than those of groups with high NLR and PLR values.^23^ Thus, based on these findings, PLR and NLR may be used to effectively predict the short-term efficacy of platinum-containing neoadjuvant chemotherapy regimens. However, some previous results contradict these findings. For example, a retrospective analysis pointed out that compared with low PLR, high PLR was significantly associated with a higher pCR rate in breast cancer treated with a taxane, anthracycline, and cyclophosphamide neoadjuvant chemotherapy regimen.^24^ This difference could be attributable to differences in the age of the patients, tumor stage, and other baseline characteristics, as well as differences in the cutoff value for NLR and PLR and the chemotherapy scheme. Meanwhile, we also focused on the relationship between dose intensity and side effects of TEL regimen. Through the analysis of the efficacy and side effects of combination chemotherapy, we finally decided to use this dose as the chemotherapy dose, which was also discussed in detail in our previous randomized control trial (RCT).^5^ Based on the published results and the data of this study, the use of TEL regimen with current dose intensity may increase the proportion of III-IV grade side effects such as leukopenia, neutropenia, anemia, and thrombocytopenia, but it is generally tolerable.^5,6^ In the present study, in addition to NLR and PLR being predictors of the pCR rate, NLR was found to be an independent predictor of EFS and OS. That is, better EFS and OS were recorded for patients with low NLR and PLR values. Thus, these 2 indicators can effectively predict the long-term efficacy of platinum-containing neoadjuvant therapy. Accordingly, studies on gastric cancer, oral tongue squamous cell carcinoma, liver cancer, and other tumors have also found that PLR and NLR were independent risk factors for prognosis, with the prognosis of low PLR/NLR groups being significantly better than that of high PLR/NLR groups.^25-28^ Further, the combination of NLR and PLR was also found to be an independent prognostic factor for predicting overall survival.^25-28^ All these findings confirm the present results and imply that PLR and NLR are closely related to tumor prognosis after platinum-containing neoadjuvant regimens.

With regard to studies that have explored the predictive ability of NLR and PLR in breast cancers, there is plenty of evidence to support their benefits for platinum-containing regimens, as well as regimens without platinum. For example, in a study on 273 patients with breast cancer who received taxane and anthracycline as the main chemotherapy agents, the NLR of patients who did not achieve pCR after neoadjuvant chemotherapy was significantly higher than that of those who achieved pCR (*P* = .016), with the RFS *(P* = .016) and OS (*P* = .038) of the high NLR group being significantly lower than those of the low NLR group.^29^ A study of 1174 cases of liver cancer found that the 5-year survival rate of patients with PLR < 150 was 71.8%, and this was significantly better than the rate of 57.2% in patients with PLR > 150 (*P* < .001).^30^ Further, OS analysis of 140 cases of melanoma showed that the OS of the low PLR group was 34 months and the OS of the high PLR group was 17 months (hazard ratio: 0.436, 95% CI, 0.291-0.652, *P* < .001).^31^ In a study on TNBC, the 5-year OS of patients with NLR > 2.65 was lower than that of patients with NLR ≤ 2.65 (*P* = .034), and the 5-year OS of patients with PLR > 190.9 was also lower than that of patients with PLR ≤ 190.9 (*P* = .032).^32^ Further, in a retrospective study on metastatic TNBC, in a cohort of 57 patients who received the taxane-carboplatin or carboplatin-capecitabine regimen, those with NLR < 2.5 had significantly better PFS (*P* < .001) and OS (*P* = .01) than those with NLR ≥ 2.5.^33^ Further, PFS was longer in the PLR < 200 group than in the PLR ≥ 200 group (*P* < .001), and NLR ≥ 2.5 was independently associated with poorer PFS (*P* = .004) and OS (*P* = .038).^33^ These findings further confirm that PLR and NLR can be used to predict the long-term efficacy of platinum drugs for the treatment of TNBC.

In addition to PLR and NLR, certain other immune cells have also been found to be associated with the prognosis of breast cancer. For example, high levels of tumor-infiltrating lymphocytes (TILs) have a good prognostic relationship with malignant tumors, such as breast cancer, and this has been widely confirmed by multiple studies.^34-39^ Further, some scholars found that PLR in patients with TNBC was significantly positively correlated with the population of Treg cells in the tumor area (*P* = .049). Therefore, high PLR might be associated with immunosuppression. In addition, combined analysis of PLR and TILs showed that high TIL levels and low PLR levels were associated with the highest values of distant metastasis-free survival (*P* = .011) and OS (*P* = .004).^32^ The present study did not investigate TIL levels, but in the future, it would be interesting to explore the combined prognostic significance with the addition of other immune cells.

There are some limitations of this study that need to be mentioned. First, there is no clear definition of the cutoff values of NLR and PLR in the literature. In this study, we used the mean value as the cutoff, and both indicators had good predictive value. Still, the optimal cutoff value needs to be determined by further research. A second limitation is the small sample size, so future research on larger cohorts is required to confirm the findings.

To conclude, this study reports for the first time the independent predictive value of PLR and NLR for early TNBC treated with a neoadjuvant regimen comprising taxane and anthracycline combined with lobaplatin and provides further reference for the selection of clinical chemotherapy regimens, avoiding the side effect of chemotherapy, and preventing the occurrence of disease progression.

These indicators are easy to assess, and in the future, they could be combined with PI3KCA, BRCA1/2, and other markers in order to improve prognosis and treatment accuracy in patients with TNBC. In our next study, we plan to explore the potential of adding other markers to improve the efficiency of prognosis prediction and the accuracy of treatment regimens for breast cancer.

## Supplementary Material

Supplementary material is available at *The Oncologist* online.

oyae025_suppl_Supplementary_Figures_1

oyae025_suppl_Supplementary_Tables_1

oyae025_suppl_Supplementary_Tables_2

## Data Availability

The data underlying this article will be shared on reasonable request to the corresponding author.

## References

[1] Perou CM , Sørlie T, Eisen MB, et al. Molecular portraits of human breast tumours. Nature. 2000;406(6797):747-752. 10.1038/35021093 10963602

[2] Berrada N , Delaloge S, André F. Treatment of triple-negative metastatic breast cancer: toward individualized targeted treatments or chemosensitization? Ann Oncol. 2010;21(Suppl 7):vii30-vii35. 10.1093/annonc/mdq279 20943632

[3] Huang M , O’Shaughnessy J, Zhao J, et al. Association of pathologic complete response with long-term survival outcomes in triple-negative breast cancer: a meta-analysis. Cancer Res. 2020;80(24):5427-5434. 10.1158/0008-5472.CAN-20-1792 32928917

[4] Dent R , Rugo HS. Most neoadjuvant chemotherapy for triple-negative breast cancer should include platinum. Lancet Oncol. 2021;22(1):27-28. 10.1016/S1470-2045(20)30747-6 33387495

[5] Wu X , Tang P, Li S, et al. A randomized and open-label phase II trial reports the efficacy of neoadjuvant lobaplatin in breast cancer. Nat Commun. 2018;9(1):832. 10.1038/s41467-018-03210-2 29483583 PMC5827032

[6] Yan W , Wu X, Wang S, et al. Lobaplatin-based neoadjuvant chemotherapy for triple-negative breast cancer: a 5-year follow-up of a randomized, open-label, phase II trial. Ther Adv Med Oncol. 2022;14:17588359221107111. 10.1177/17588359221107111 35769355 PMC9234826

[7] Zhou Y , Heitmann JS, Clar KL, et al. Platelet-expressed immune checkpoint regulator GITRL in breast cancer. Cancer Immunol Immunother. 2021;70(9):2483-2496. 10.1007/s00262-021-02866-y 33538861 PMC8360840

[8] Hinterleitner C , Zhou Y, Tandler C, et al. Platelet-expressed TNFRSF13B (TACI) predicts breast cancer progression. Front Oncol. 2021;11:642170. 10.3389/fonc.2021.642170 33816291 PMC8010255

[9] Zhou Y , Heitmann JS, Kropp KN, et al. Regulation of platelet-derived ADAM17: a biomarker approach for breast cancer? Diagnostics (Basel). 2021;11(7):1188. 10.3390/diagnostics11071188 34208863 PMC8305148

[10] Mendoza-Almanza G , Burciaga-Hernández L, Maldonado V, Melendez-Zajgla J, Olmos J. Role of platelets and breast cancer stem cells in metastasis. World J Stem Cells. 2020;12(11):1237-1254. 10.4252/wjsc.v12.i11.1237 33312396 PMC7705471

[11] Liu S , Fang J, Jiao D, Liu Z. Elevated platelet count predicts poor prognosis in breast cancer patients with supraclavicular lymph node metastasis. Cancer Manag Res. 2020;12:6069-6075. 10.2147/CMAR.S257727 32765104 PMC7381764

[12] Xiao Y , Cong M, Li J, et al. Cathepsin C promotes breast cancer lung metastasis by modulating neutrophil infiltration and neutrophil extracellular trap formation. Cancer Cell. 2021;39(3):423-437.e7. 10.1016/j.ccell.2020.12.012 33450198

[13] Ma W , Zhang P, Qi J, et al. Prognostic value of platelet to lymphocyte ratio in hepatocellular carcinoma: a meta-analysis. Sci Rep. 2016;6:35378. 10.1038/srep35378 27739490 PMC5064312

[14] Cummings M , Merone L, Keeble C, et al. Preoperative neutrophil:lymphocyte and platelet:lymphocyte ratios predict endometrial cancer survival. Br J Cancer. 2015;113(2):311-320. 10.1038/bjc.2015.200 26079303 PMC4506386

[15] Huszno J , Kolosza Z, Mrochem-Kwarciak J, Rutkowski T, Skladowski K. The role of neutrophil-lymphocyte ratio, platelet-lymphocyte ratio, and platelets in the prognosis of metastatic renal cell carcinoma. Oncology. 2019;97(1):7-17. 10.1159/000498943 31048577

[16] Ethier JL , Desautels D, Templeton A, Shah PS, Amir E. Prognostic role of neutrophil-to-lymphocyte ratio in breast cancer: a systematic review and meta-analysis. Breast Cancer Res. 2017;19(1):2. 10.1186/s13058-016-0794-1 28057046 PMC5217326

[17] Asano Y , Kashiwagi S, Onoda N, et al. Predictive value of neutrophil/lymphocyte ratio for efficacy of preoperative chemotherapy in triple-negative breast cancer. Ann Surg Oncol. 2016;23(4):1104-1110. 10.1245/s10434-015-4934-0 26511266 PMC4773470

[18] Chae S , Kang KM, Kim HJ, et al. Neutrophil-lymphocyte ratio predicts response to chemotherapy in triple-negative breast cancer. Curr Oncol. 2018;25(2):e113-e119. 10.3747/co.25.3888 29719435 PMC5927790

[19] Cuello-López J , Fidalgo-Zapata A, López-Agudelo L, Vásquez-Trespalacios E. Platelet-to-lymphocyte ratio as a predictive factor of complete pathologic response to neoadjuvant chemotherapy in breast cancer. PLoS One. 2018;13(11):e0207224. 10.1371/journal.pone.0207224 30427884 PMC6235359

[20] Gong Z , Xin R, Li L, Lv L, Wu X. Platelet-to-lymphocyte ratio associated with the clinicopathological features and prognostic value of breast cancer: a meta-analysis. Int J Biol Markers. 2022;37(4):339-348. 10.1177/03936155221118098 35971299

[21] Lusho S , Durando X, Mouret-Reynier MA, et al. Platelet-to-lymphocyte ratio is associated with favorable response to neoadjuvant chemotherapy in triple negative breast cancer: a study on 120 patients. Front Oncol. 2021;11:678315. 10.3389/fonc.2021.678315 34367964 PMC8331686

[22] McLaren PJ , Bronson NW, Hart KD, et al. Neutrophil-to-lymphocyte and platelet-to-lymphocyte ratios can predict treatment response to neoadjuvant therapy in esophageal cancer. J Gastrointest Surg. 2017;21(4):607-613. 10.1007/s11605-016-3351-4 28083838

[23] Liu M , Feng Y, Zhang Y, Liu H. Evaluation of neutrophil-lymphocyte ratio and platelet-lymphocyte ratio on predicting responsiveness to neoadjuvant chemoradiotherapy in locally advanced rectal cancer patients. Biomed Res Int. 2022;2022:3839670. 10.1155/2022/3839670 36212713 PMC9534654

[24] Jin X , Wang K, Shao X, Huang J. Prognostic implications of the peripheral platelet-to-lymphocyte ratio and neutrophil-to-lymphocyte ratio in predicting pathologic complete response after neoadjuvant chemotherapy in breast cancer patients. Gland Surg. 2022;11(6):1057-1066. 10.21037/gs-22-244 35800742 PMC9253186

[25] Wang B , Liu J, Zhong Z. Prediction of lymph node metastasis in oral tongue squamous cell carcinoma using the neutrophil-to-lymphocyte ratio and platelet-to-neutrophil ratio. J Clin Lab Anal. 2021;35(6):e23684. 10.1002/jcla.23684 33942387 PMC8183927

[26] Yu JI , Park HC, Yoo GS, et al. Clinical importance of the absolute count of neutrophils, lymphocytes, monocytes, and platelets in newly diagnosed hepatocellular carcinoma. Sci Rep. 2021;11(1):2614. 10.1038/s41598-021-82177-5 33510378 PMC7844216

[27] Schobert IT , Savic LJ, Chapiro J, et al. Neutrophil-to-lymphocyte and platelet-to-lymphocyte ratios as predictors of tumor response in hepatocellular carcinoma after DEB-TACE. Eur Radiol. 2020;30(10):5663-5673. 10.1007/s00330-020-06931-5 32424595 PMC7483919

[28] Hirahara T , Arigami T, Yanagita S, et al. Combined neutrophil-lymphocyte ratio and platelet-lymphocyte ratio predicts chemotherapy response and prognosis in patients with advanced gastric cancer. BMC Cancer. 2019;19(1):672. 10.1186/s12885-019-5903-y 31286873 PMC6615151

[29] Bae SJ , Ahn SG, Ji JH, et al. Prognostic value of neutrophil-to-lymphocyte ratio and early standardized uptake value reduction in patients with breast cancer receiving neoadjuvant chemotherapy. J Breast Cancer. 2022;25(6):485-499. 10.4048/jbc.2022.25.e44 36479600 PMC9807322

[30] Yang Y , Wang MC, Tian T, et al. A high preoperative platelet-lymphocyte ratio is a negative predictor of survival after liver resection for hepatitis B virus-related hepatocellular carcinoma: a retrospective study. Front Oncol. 2020;10:576205. 10.3389/fonc.2020.576205 33178607 PMC7597590

[31] Qi Y , Zhang Y, Fu X, et al. Platelet-to-lymphocyte ratio in peripheral blood: A novel independent prognostic factor in patients with melanoma. Int Immunopharmacol. 2018;56:143-147. 10.1016/j.intimp.2018.01.019 29414644

[32] Huszno J , Kolosza Z. Prognostic value of the neutrophil-lymphocyte, platelet-lymphocyte and monocyte-lymphocyte ratio in breast cancer patients. Oncol Lett. 2019;18(6):6275-6283. 10.3892/ol.2019.10966 31788105 PMC6865674

[33] Vernieri C , Mennitto A, Prisciandaro M, et al. The neutrophil-to-lymphocyte and platelet-to-lymphocyte ratios predict efficacy of platinum-based chemotherapy in patients with metastatic triple negative breast cancer. Sci Rep. 2018;8(1):8703. 10.1038/s41598-018-27075-z 29880896 PMC5992181

[34] Denkert C , von Minckwitz G, Darb-Esfahani S, et al. Tumour-infiltrating lymphocytes and prognosis in different subtypes of breast cancer: a pooled analysis of 3771 patients treated with neoadjuvant therapy. Lancet Oncol. 2018;19(1):40-50. 10.1016/S1470-2045(17)30904-X 29233559

[35] Wang S , Sun J, Chen K, et al. Perspectives of tumor-infiltrating lymphocyte treatment in solid tumors. BMC Med. 2021;19(1):140. 10.1186/s12916-021-02006-4 34112147 PMC8194199

[36] Loi S , Michiels S, Adams S, et al. The journey of tumor-infiltrating lymphocytes as a biomarker in breast cancer: clinical utility in an era of checkpoint inhibition. Ann Oncol. 2021;32(10):1236-1244. 10.1016/j.annonc.2021.07.007 34311075

[37] Laumont CM , Banville AC, Gilardi M, Hollern DP, Nelson BH. Tumour-infiltrating B cells: immunological mechanisms, clinical impact and therapeutic opportunities. Nat Rev Cancer. 2022;22(7):414-430. 10.1038/s41568-022-00466-1 35393541 PMC9678336

[38] Mahmoud SM , Paish EC, Powe DG, et al. Tumor-infiltrating CD8+ lymphocytes predict clinical outcome in breast cancer. J Clin Oncol. 2011;29(15):1949-1955. 10.1200/JCO.2010.30.5037 21483002

[39] Brummel K , Eerkens AL, de Bruyn M, Nijman HW. Tumour-infiltrating lymphocytes: from prognosis to treatment selection. Br J Cancer. 2023;128(3):451-458. 10.1038/s41416-022-02119-4 36564565 PMC9938191

