# Supplementary figures and images for: Two Hematological Markers Predicting the Efficacy and Prognosis of Neoadjuvant Chemotherapy Using Lobaplatin Against Triple-Negative Breast Cancer
Source: Oncologist. 2024 Mar 2;29(5):e635–42. doi: 10.1093/oncolo/oyae025 (PMC11067820; doi:10.1093/oncolo/oyae025)

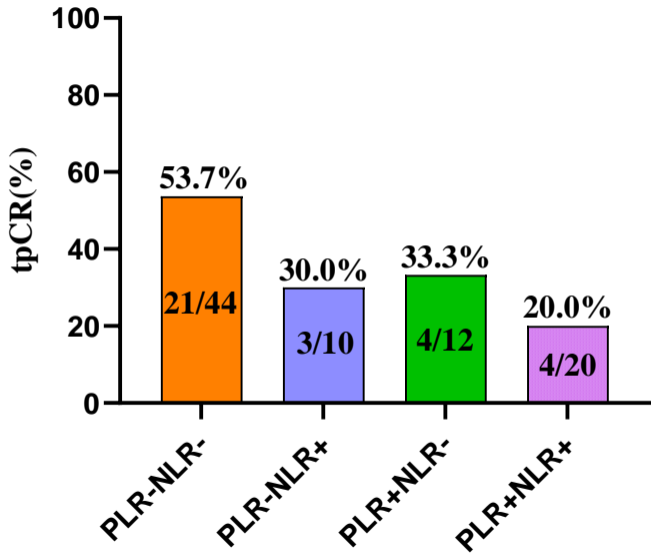

Supplement: oyae025_suppl_Supplementary_Figures_1 [file oyae025_suppl_supplementary_figures_1.pdf]
