# Supplementary material for: Two Hematological Markers Predicting the Efficacy and Prognosis of Neoadjuvant Chemotherapy Using Lobaplatin Against Triple-Negative Breast Cancer
Source: Oncologist. 2024 Mar 2;29(5):e635–42. doi: 10.1093/oncolo/oyae025 (PMC11067820; doi:10.1093/oncolo/oyae025)
Supplement: oyae025_suppl_Supplementary_Tables_1 [file oyae025_suppl_supplementary_tables_1.docx]

Supplementary Table 1. Univariate Analysis for EFS.

| Variable | HR(95%CI) | P |
| --- | --- | --- |
| Age(≥45 vs.<45) | 0.492(0.139-1.744) | 0.272 |
| her2(0 vs.1/2) | 0.940(0.200-4.428) | 0.938 |
| T(3/4 vs.1/2) | 0.847(0.107-6.692) | 0.875 |
| N(2/3 vs. 0/1) | 8.799(2.465-31.408) | 0.001 |
| ki67(≥30% vs.<30%) | 0.971(0.251-3.758) | 0.966 |
| PLR(>145.71 vs.<145.71) | 1.861(0.537-6.443) | 0.327 |
| NLR(>2.74 vs.<2.74) | 5.946(1.526-23.169) | 0.010 |
