# Supplementary material for: Two Hematological Markers Predicting the Efficacy and Prognosis of Neoadjuvant Chemotherapy Using Lobaplatin Against Triple-Negative Breast Cancer
Source: Oncologist. 2024 Mar 2;29(5):e635–42. doi: 10.1093/oncolo/oyae025 (PMC11067820; doi:10.1093/oncolo/oyae025)
Supplement: oyae025_suppl_Supplementary_Tables_2 [file oyae025_suppl_supplementary_tables_2.docx]

Supplementary Table 2. Univariate Analysis for OS.

| Variable | HR(95%CI) | P |
| --- | --- | --- |
| Age(≥45 vs.<45) | 0.734(0.183-2.937) | 0.662 |
| Her2(0 vs.1/2) | 1.229(0.248-6.093) | 0.800 |
| T(3/4 vs.1/2) | 1.175(0.144-9.560) | 0.880 |
| N(2/3 vs. 0/1) | 16.518(3.321-82.159) | 0.001 |
| ki67(≥30% vs.<30%) | 1.320(0.266-6.552) | 0.734 |
| PLR(>145.71 vs.<145.71) | 1.850(0.462-7.414) | 0.385 |
| NLR(>2.74 vs.<2.74) | 7.803(1.565-38.907) | 0.012 |
